# Exposure to HIV risks among young people who use drugs (YPUD) in three cities in Vietnam: time to develop targeted interventions

**DOI:** 10.1186/s12954-020-00357-4

**Published:** 2020-02-24

**Authors:** Laurent Michel, Linh T. Nguyen, An K. Nguyen, John P. Ekwaru, Didier Laureillard, Nicolas Nagot, Olivier Phan, Oanh T. H. Khuat

**Affiliations:** 1 CESP/Inserm 1018, Centre Pierre Nicole, French Red Cross, Paris, France; 2 Supporting Community Development Initiative, 240 Mai Anh Tuan Street, Thanh Cong Ward, Ba Dinh District, Ha Noi, Vietnam; 3 School of Public Health, University of Alberta, Edmonton, Canada; 4 Infectious Diseases Department, Caremeau University Hospital, Nîmes, France; 5 Pathogenesis and control of chronic infections, Inserm, Etablissement Français du Sang, University of Montpellier, Montpellier, France

**Keywords:** Young people, Methamphetamines, HIV, Mental health, Sexual risk, Drug use

## Abstract

**Introduction:**

The aim of this study is to identify the profiles of young people who use drugs (YPUD) and their exposure to HIV risks in the 3 main cities of Vietnam, Haiphong, Hanoi, and Ho Chi Minh City (HCMC), in order to design a community-based intervention to prevent HIV.

**Methods:**

A survey using respondent-driven sampling (RDS) was conducted among YPUD aged 16–24. Participants were eligible if they reported drug use, confirmed by a urine test. After obtaining informed consent, they were screened for HIV/HCV and assessed using face-to-face questionnaires and self-report. A cluster analysis was conducted, taking into account risk behaviors and confirmed HIV-positive status.

**Results:**

Seven hundred and three YPUD aged 16–24 were recruited between October 2016 and February 2017, 584 of whom were included in the final analysis. Median age was 21 (17.7, 23.0); 79% were male, 18% female, and 2% transgender. Methamphetamines use was reported by 77%, followed by cannabis (51%) and heroin (17%); polydrug use was common; 15% had “ever” injected drugs. HIV prevalence was 7%. Among all participants, 48% reported non-consistent condom use and 1% reported needle/syringe sharing during the previous month. Four distinct profiles of HIV risk behaviors were identified: The high multiple-risk group mixed unsafe drug use with unsafe sexual practices and had higher prevalence of HIV; the second group practiced high-risk sex with non-consistent condom combined with methamphetamine use; the third group was a moderate-risk group with limited unsafe sexual practices; and the fourth was considered at “low-risk” as reportedly, most never had sex and never injected. The highest risk group included more female YPUD, living in HCMC, who used heroin and had unsafe sex with their regular partners. The second high-risk group included most of the MSM and all transgender people and frequently reported mental health disorders.

**Conclusions:**

The profiles of YPUD who are at risk of HIV vary according to age, location, and population group. Injecting YPUD are the most exposed to risk and need immediate attention. Sexual exposure to HIV is very common. Mental health is a major concern. Interventions need to be integrated in a differentiated but holistic approach.

## Background

### HIV—the global context

According to WHO, more than 4 in 10 people worldwide were younger than 25 in 2016 and 16% of the total population was aged 15–24. This population is the most affected by the global epidemic of HIV. Thus, in 2013, young people aged 15–24 accounted for an estimated 35% of all new HIV infections worldwide in people over 15 [1]. Drug use is closely associated with HIV exposure, through unsafe injection practices or sexual risk behaviors associated with drug use (e.g., lack of inhibitions and loss of control during sexual intercourse, sex work, risky sexual practices). Both early (12–14) and late adolescence (15–17) years old are a critical risk period for the initiation of substance use, which tends to peak among young people aged 18–25 [2]. In East and Southeast Asia, 24.9% of the people who inject drugs (PWID) are younger than 25 [3].

Drug use during adolescence poses a high risk because it may seriously impact neurodevelopment, causing cognitive, social, and psychiatric consequences [2]. Furthermore, services for drug users are rarely designed to meet the specific needs of this population and some even restrict access to those under 16 or 18 [1]. And lastly, young people who use drugs are more likely to have experienced early childhood adversity such as psychiatric disorders in their family or an unstable environment, leading to marginalization and risk behaviors [4].

According to WHO and UNODC, screening and brief interventions are effective in preventing progression to substance use disorders for young people who have initiated substance use. But family context, socioeconomic environment, and mental health condition need to be addressed [2].

### HIV in Vietnam

Vietnam is a country of 96 million people, facing a concentrated HIV epidemic among three populations defined by high levels of HIV-risk behaviors: people who use drugs (PWID), men who have sex with men (MSM), and female sex workers (FSW) and their clients. An estimated 256,000 people were living with HIV in Vietnam in 2014 with around 14,000 new cases reported yearly from 2010 to 2013. The percentage of new HIV cases among PWID is decreasing, whereas sexual transmission of HIV has increased among MSM and in both male clients of FSWs and low-risk women whose partners are infected [5]. Seventy-nine percent of PLHIV are between 20 and 39 years old, and 33.9% are under 29 (Annual Report of the HIV Prevention and Control Program 2013 and Plan for 2014, Ministry of Health, 2014). Disaggregated data on 15–24-year-old drug users are lacking and this population is poorly represented in the existing surveys. Drug use in Vietnam aggressively shifted since the 1990s from opium to heroin, and crystal methamphetamine (“ice”) use has been rapidly increasing since the early 2000s [5]. Methamphetamine use is associated with different high-risk behaviors, including unprotected sex with multiple partners, marathon sex, and polydrug use [6]. In the city of Haiphong in 2016, among 1336 PWID (heroin) older than 18 and recruited through respondent-driven sampling, 51% reported current methamphetamine use, which was generally smoked (only a few PWID reported injecting this drug). Methamphetamine use was associated with sexual risk behaviors among HIV-positive PWID. The median age of the population sample was 39 (9 SD) and only 60 (4.3%) were aged 18 to 24 [7].

The aim of this study is to identify those among a sample of young drug users (16–24) in 3 major cities of Vietnam (Hanoi, Haiphong, and HCMC), who are most exposed to HIV, by examining their sexual- and drug use-risk behaviors. The objective is to design a tailored community-based intervention for HIV prevention adapted to their specific needs.

## Material and methods

### Recruitment procedure

Considering the difficulty of reaching YPUD in the community, participants were recruited using two procedures. The first was a respondent-driven sampling (RDS) strategy [8, 9] and the second was a system of peer recruitment [10]. It was expected to recruit 600 participants, 200 in each city. Initially “seeds” were selected from community-based organizations (CBO) or key population networks, representing the diversity of sub-key populations (MSM, transgender, sex workers, and regular young people who use drugs), to ensure a diversity of ages and of living arrangements. Each seed first participated in study procedures and then was given three coupons to distribute to potentially eligible participants. These pre-selected “seed” participants were believed to have a wide network and able to reach out to other young drug users who are likely to meet the recruiting criteria. Persons presenting coupons at the research site were invited to participate in the study; after participating, they were given coupons to recruit new participants. RDS recruiting continued until the target sample size was reached. An additional recruitment procedure was introduced when the RDS numbers were diminishing, and some direct recruitment by CBO members was initiated. Thus, the number of coupons distributed was increased until the expected number of participants was reached. Participants received VND 150,000 ($7.50 USD) for their participation and VND 50,000 ($2.50 USD) for each YPUD they helped recruit and all participants received VND 100,000 for coming back to get their screening results.

The inclusion criteria were (1) aged 16 to 24; (2) urine-tested positive to at least 1 of 4 illicit drugs, heroin, methamphetamine, cannabis, and ecstasy; (3) agreement to provide written consent to participate in the study; and (4) the ability to fully understand the study’s purpose and questions and sign the consent form.

The study was approved by the Institutional Review Board of the Institute for Social Development and Studies (Hanoi).

### Data collection

After eligibility was confirmed and informed consent was obtained, a structured questionnaire was administered by a trained interviewer. Data were collected on socio-demographic and family characteristics, drug use, risk behaviors related to drug use, access to health services, alcohol use, tattooing, internet use, and knowledge about HIV and HCV. Depression was assessed using the Adolescent Depression Rating Scale (ADRS) [11]. Psychotic experiences were assessed by a 6-item sub questionnaire of the “positive” subscale from the Community Assessment of Psychic Experience questionnaire (CAPE), initially designed for measurement of psychosis proneness [12, 13] (we used 2 items from the “bizarre ideations” dimension, 2 items from the “perceptual anomalies” dimension, and 2 items from the “delusional ideations” dimension, the score ranging from 6 to 24). The sexual behaviors and adverse childhood experience [14] were assessed through a self-completion questionnaire (ACE questionnaire). All participants were screened for HIV and HCV (by providing a blood sample).

### Statistical analysis

To identify profiles of respondents with specific patterns of HIV-transmission risk, multiple correspondence analysis was first carried out on the active variables. Values of the main dimensions obtained from multiple correspondence analysis (MCA) were then used as continuous variables in hierarchical ascendant classification. All analysis was carried out using SAS software (SAS Institute, Cary, NC, USA). Multiple correspondence analysis was carried out using SAS procedure CORRESP and hierarchical ascendant classification was carried out using the SAS procedure CLUSTER. All statistical analysis results, except sample characteristics, were weighted using RDS-II (Volz-Heckathorn) weights [15].

Inactive variables used to label profile clusters in terms of HIV-transmission risk included “ever” injected drugs using needles/syringes already used, “ever” sharing with someone else used needles/syringes, not always using condoms during sexual intercourse, and being HIV positive. Descriptive variables included in the final analysis to characterize the different subgroups of YPUD with HIV-infection risk included socio-demographic characteristics including city of residence, current and past patterns of drug and alcohol use (with the exception of drug-related risk behaviors), sexual behaviors (with the exception of inconstant condom use), mental health status, and tattoo.

## Results

From October 2016 to February 2017, 703 YPUD were recruited, 604 completed the survey, and 584 were eligible for data analysis (20 were identified as older than 24). Four hundred and twenty-six were recruited through RDS and 277 through peer recruitment (Table 1).

**Table 1:** Number of YPUD recruited according to the city and the recruitment procedure

City	Number of seeds	Recruitment procedure		Recruitment sample size *N* (%)		
		RDS	Select peer recruitment	Total recruited	Completed the survey	Eligible for data analysis
Hanoi	23	105	97	225	179	168 (28.8)
Haiphong	19	158	66	243	217	213 (36.5)
Ho Chi Minh City	30	91	114	235	208	203 (34.8)
Total	**72**	**354**	**277**	**703**	**604**	**584**

Participants were mainly male (79%), and nearly a third of them (31%) did not have permanent accommodation. Most had already left school (78%), spending most of their time with peers (45%) rather than with family members (35%). Based on self-reports and urine testing, methamphetamines are the most commonly used drugs, 77% and 71%, respectively. Overall, only 15% of YPUD had ever injected drugs, mainly heroin, with a low rate of self-reported syringe/needle sharing (1% in the past month). But in HCMC, 30% reported “ever” injecting drugs and among them, 23% “ever” used non-clean needles/syringes (none in Haiphong and Hanoi), and 21% “ever” shared their own needles/syringes (none in Haiphong and 1 YPUD in Hanoi). The most frequent motivation for methamphetamine use was reported to be relief from sadness and loneliness (48% of the total sample). Most of the YPUD (62%) considered that they can control their drug use and set limits on use, but half (50%) reported that they often felt bad about their drug use, and 30% said that they needed help, however just 9% of them had been in contact with health professionals.

Half of the sample presented with depression as measured by the ADRS and 13% were exhibiting “often” or “nearly always” delusional ideas, 20% “often” or “nearly always” expressed bizarre ideations and 6% “often” or “nearly always” experienced some hallucinations (CAPE). Eighteen percent answered “yes” when asked about a perceived need for mental health treatment. Among those who “ever” had sex (71%), only one third always used a condom during sexual intercourse. Nearly 1/10 reported having been forced to have sex with someone: 7% (31) of men, 19% (21) of females, and 27% (4) of transgenders. Knowledge on HIV and HCV transmission was limited: 15% did not know that it can be transmitted through sharing of contaminated syringe/needle, 5% did not know HIV could be transmitted through unprotected sex, and 76% reported that they did not know how HCV is transmitted. Among this population sample, 37 (6.3%) were HIV positive (26/460 male, 10/109, female and 1/15 transgender; 11/37 reported having sex with someone of the same sex), 1 was 16 years old, 10 were aged between 19 and 22, and 26 were 23 or older; 30 were in Ho Chi Minh City (including the 10 female and 10/11 of the YPUD reporting having sex with someone of the same sex), 6 in Hanoi, and 1 in Haiphong. Among YPUD who were HIV positive, 19 reported “ever” injecting drugs, 22 reported that they had not been screened for HIV prior to the study, 15 said that they had already been screened, but 6 said that they had no knowledge of the result. Altogether, 28/37 of HIV positive YPUD did not know their sero-status prior to the study. Among HIV-positive YPUD, 19 (51.3%) “ever” injected drugs, 5 “ever” injected drugs with syringes/needles already used by someone else and 5 “ever” shared their syringes/needles with someone else, including 4 during their last injection (none of them had at the time of sharing been tested for HIV).

**Fig. 1:**
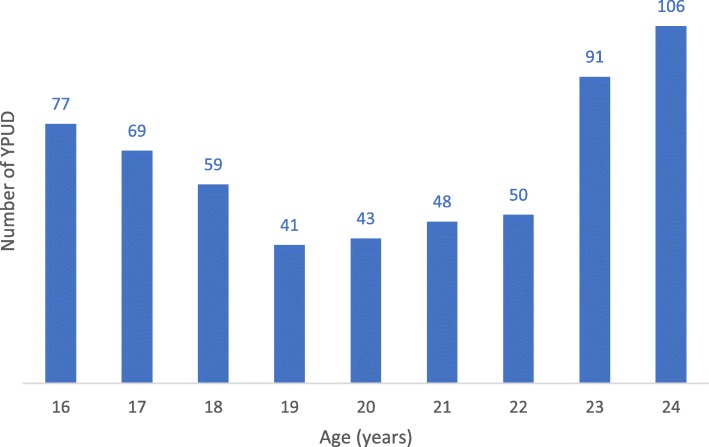
Number of YPUD according to age (*n* = 584)

The main characteristics of the sample are presented in Table 2 and Fig. 1 that presents the age distribution.

**Table 2:** Main characteristics of the YPUD sample (*n* = 584)

	*N* (%)
Gender	
Male	460 (78.8)
Female	109 (18.7)
Transgender	15 (2.6)
Age (median, IQR)	21 (17.75, 23.0)
Have an ID card	419 (71.7)
Currently at school	130 (22.3)
Parents	
Married/living together	331 (56.7)
Mother deceased	43 (7.4)
Father deceased	94 (16.1)
Family context: past history of	
Drug use in the family	134 (22.9)
Mental health problem	33 (5.7)
HIV	34 (5.8)
Prison or rehabilitation center	185 (31.7)
Living	
On the street	12 (2.1)
In a rented house	143 (24.5)
In family or own house	401 (68.7)
In workplace	6 (1.0)
Somebody else’s house	14 (2.4)
Other/no answer	8 (1.4)
Generating income/having a job	340 (58.2)
Marital status	
Single	501 (85.8)
Married/living in a couple	71 (12.2)
Divorced or widowed	12 (2.1)
Having a child(dren)	79 (13.5)
Age (years) at first use of drugs (median, IQ)	16.0 (15.0, 19.0)
Current use of drugs (last 3 months)	
Heroin	101 (17.3)
Cannabis	299 (51.2)
Methamphetamines	448 (76.7)
Ecstasy	56 (9.6)
Ketamine	46 (7.9)
Drug most commonly used (last 3 months)	
Heroin	65 (11.1)
Cannabis	200 (34.2)
Methamphetamines	306 (52.4)
Ecstasy	8 (1.4)
Ketamine	5 (0.9)
Urine test	
Opiates	105 (18.0)
Methamphetamines	414 (70.9)
Cannabis	252 (43.2)
Ecstasy	16 (2.7)
Ever used methadone	17 (2.9)
Ever injected drugs	87 (14.9)
Heroin	83 (14.2)
Methamphetamines	9 (1.5)
Methamphetamine use	
Less than once a week	159 (27.2)
Once a week to several times a week	209 (35.8)
Everyday	98 (16.8)
Ever received a treatment	67 (11.5)
Alcohol use (last 6 months)	
Never	174 (29.8)
Monthly or less	137 (23.5)
2–4 times a month	132 (22.6)
2–3 times a week	78 (13.4)
4 times a week or more	63 (10.8)
Smoking cigarettes every day	425 (72.8)
Has a tattoo	278 (47.6)
Mean time (h daily) spent on internet (median, IQ)	4.0 (2.0, 8.0)
Mean time (h daily) spent playing video games (median, IQ)	2.0 (0.0, 4.0)
Depression (ADRS)	
Score 0–3	290 (49.7)
Score ≥ 4–< 8 (moderate depression)	214 (36.7)
Score ≥ 8 (severe depression)	80 (13.7)
Psychotic experience (CAPE) (median, IQ)	8.0 (7.0, 10.0)
Ever tested	
For HIV	226 (38.7)
For HCV	64 (11.0)
HIV/HCV sero-status	
HIV positive	37 (6.3)
HCV positive	55 (9.4)
HIV/HCV co-infection	19 (3.3)
Ever had sex	
Yes	412 (70.5)
No	163 (27.9)
No answer	9 (1.5)
Number of sexual partners during last 3 months (*n* = 412)	
0	72 (17.5)
1	192 (46.6)
2 or more	148 (35.9)
Less likely to use a condom under the influence of drugs	184 (31.5)
More sexual activity after using drugs	189 (32.4)
Sex with person of the same sex	81 (13.9)
Male	63 (10.8)
Female	5 (0.9)
Transgender	13 (2.2)
Being paid for sexual intercourse	84 (14.4)
Male	44 (7.5)
Female	33 (5.7)
Transgender	7 (1.2)
Have ever been forced to have sex with someone	56 (9.6)

### Profiles of HIV-infection risk

From the cluster analysis, 4 profiles of YPUD at risk of HIV-infection have emerged. These profiles were constructed using self-reported variables on the sharing of needles and syringes, condom use, and on independently confirmed HIV sero-status. The 4 profiles are (1) multiple high risks, (2) high sexual risks, (3) moderate risks, and (4) low risks.

Distribution of risk variables by profile is presented in Table 3 and a comparison of the descriptive variables among the different population profiles is presented in Table 4. A summary of the characteristics of the different profiles of HIV exposure and risks is presented in Table 5.

**Table 3:** Distribution of risk variables by profile (*n* = 584)

	Overall		Profile 1, high multiple risks (*n* = 130)		Profile 2, high sexual risks (*n* = 208)		Profile 3, moderate risks (*n* = 72)		Profile 4, low risks (*n* = 174)		
Variable	*N*	Weighted %	*n*	Weighted %	*n*	Weighted %	*n*	Weighted %	*n*	Weighted %	*p* value
Ever injected drugs using used needles											
Yes	11	1.5	9	4.2	2	0.9	0	0.0	0	0.0	< 0.001
Injected only with own needle	37	8.2	19	16.7	16	10.3	0	0.0	2	1.0	
Never injected drugs	496	83.7	80	63.2	175	84.6	71	99.8	170	96.3	
Missing data/not applicable	40	6.7	22	15.9	15	4.2	1	0.2	2	2.7	
Ever given someone else to inject with used needles											
Yes	11	1.3	10	4.4	1	0.2	0	0.0	0	0.0	< 0.001
Injected, never give used needle	37	8.3	18	16.5	17	11.0	0	0.0	2	1.0	
Never injected drugs	496	83.7	80	63.2	175	84.6	71	99.8	170	96.3	
Missing data/not applicable	40	6.7	22	15.9	15	4.2	1	0.2	2	2.7	
Uses condoms always											
No	273	47.9	94	74.4	135	64.9	44	64.0	0	0.0	< 0.001
Yes	135	20.3	34	20.2	72	34.9	28	36.0	1	0.1	
Never had sex	163	27.6	0	0.0	0	0.0	0	0.0	163	91.2	
Missing data/not applicable	13	4.3	2	5.4	1	0.2	0	0.0	10	8.7	
HIV											
Negative	547	93.2	107	84.2	198	94.4	69	97.1	173	99.2	< 0.001
Positive	37	6.8	23	15.8	10	5.6	3	2.9	1	0.8	

Note: All percentages are weighted percentages accounting for the RDS sampling

**Table 4:** Distribution of active variables by identified risk profiles (*n* = 584)

Variable	Overall		Profile 1, high multiple risks (*n* = 130)		Profile 2, sexual risks (*n* = 208)		Profile 3, moderate risks (*n* = 72)		Profile 4, low risks (*n* = 174)		
	*N*	Weighted %	*n*	Weighted %	*n*	Weighted %	*n*	Weighted %	*n*	Weighted %	*p* value
City											
Hanoi	168	27.6	33	32.2	58	20.1	22	31.8	55	29.1	< 0.001
HCMC	203	40.3	90	63.4	61	33.6	19	26.7	33	30.1	
Haiphong	213	32.1	7	4.3	89	46.3	31	41.4	86	40.8	
Sex											
Boy	460	71.7	86	49.0	149	71.2	64	86.9	161	88.2	< 0.001
Girl	109	26.0	44	51.0	44	21.3	8	13.1	13	11.8	
Transgender	15	2.2	0	0.0	15	7.5	0	0.0	0	0.0	
Age, median (IQR)	584		130	23.0 (21.0, 24.0)	208	22.0 (20.0, 23.0)	72	20.0 (18.0, 21.0)	174	17.0 (16.0, 19.0)	< 0.001
Parents married or living together	331	57.7	67	55.7	102	48.3	47	69.0	115	64.8	0.003
Mother or father dead											
Yes	120	19.1	35	27.8	48	16.9	14	17.9	23	13.5	0.005
No	459	80.0	95	72.2	156	80.8	58	82.1	150	85.9	
Do not know/no answer	5	0.9	0	0.0	4	2.3	0	0.0	1	0.7	
Living alone or with friends	99	19.4	18	13.7	64	37.8	5	10.3	12	10.0	< 0.001
Resident registration in city											
Yes	496	78.4	104	69.7	170	78.3	69	95.3	153	80.5	< 0.001
No	81	20.2	25	30.1	37	21.7	3	4.7	16	15.2	
Do not know	7	1.4	1	0.2	1	0.0	0	0.0	5	4.3	
Live most of the time											
On the street	12	1.3	10	3.9	2	0.5	0	0.0	0	0.0	< 0.001
In a rented house	143	29.7	42	43.6	73	41.3	10	18.2	18	9.2	
In a house of family/own	401	59.7	75	47.8	117	47.7	62	81.8	147	74.9	
In workplace/somebody else house/other	27	9.1	3	4.7	15	0.9	0	0.0	9	15.8	
No answer	1	0.2	0	0.0	1	0.5	0	0.0	0	0.0	
Regular income	279	55.1	65	60.3	137	69.7	29	40.8	48	41.1	< 0.001
Family mental health history											
Yes	33	6.3	11	11.4	11	4.8	2	0.6	9	5.1	0.019
No	546	92.9	116	87.3	195	94.0	70	99.4	165	94.9	
Do not know	5	0.7	3	1.4	2	1.2	0	0.0	0	0.0	
Age of first drug use											
< 14	56	7.0	11	3.9	21	8.1	3	3.7	21	9.9	< 0.001
14–15	151	21.4	27	22.0	41	13.5	14	18.0	69	29.7	
16–17	159	27.1	21	14.2	48	22.5	25	30.8	65	42.4	
18–19	109	22.8	30	28.0	47	25.5	20	34.0	12	11.2	
20–21	69	12.9	21	13.8	39	23.1	5	8.0	4	3.6	
22+	40	8.9	20	18.0	12	7.2	5	5.4	3	3.2	
Ever used heroin	148	24.3	72	48.3	62	27.3	5	8.8	9	4.3	< 0.001
Ever used cannabis	404	59.2	34	13.5	170	80.4	51	70.6	149	77.2	< 0.001
Ever used ice	487	83.1	116	89.8	202	98.5	55	68.7	114	66.7	< 0.001
Ever used ecstasy	172	25.0	25	14.7	120	56.1	13	24.3	14	4.1	< 0.001
Ever used ketamine	145	17.8	15	5.6	103	42.3	12	18.3	15	4.7	< 0.001
Use ice											
Never used	97	16.9	14	10.2	6	1.5	17	31.3	60	33.3	< 0.001
Up to once or twice a month	128	24.8	22	20.7	48	27.7	20	26.0	38	25.3	
Only at weekend or on special occasions	31	5.1	3	2.8	14	6.7	5	6.6	9	5.1	
Weekly	209	36.3	60	44.2	84	43.9	24	32.8	41	22.4	
Every day/several times a day	98	13.1	29	17.6	50	17.4	3	1.5	16	8.8	
Other/no answer	21	3.9	2	4.4	6	3.0	3	1.8	10	5.0	
Received treatment for drug use	67	10.3	46	30.4	18	4.3	1	0.6	2	0.9	< 0.001
Used alcohol in last 6 months											
Never	174	34.8	78	63.2	29	12.2	27	44.1	40	27.0	< 0.001
Monthly or less	137	26.2	20	18.1	42	25.5	14	14.2	61	39.1	
2–4 times a month	132	20.8	16	8.7	58	31.6	16	25.3	42	20.1	
2–3 times a week	78	11.4	6	6.8	39	16.4	8	12.3	25	10.4	
4 times a week or more than	63	6.8	10	3.3	40	14.4	7	4.1	6	3.4	
Got a tattoo	278	44.4	83	52.5	114	49.8	30	39.0	51	33.3	0.001
Use drugs to enhance skills when playing videogames											
Yes	130	17.0	16	6.7	60	25.5	14	17.7	40	18.0	< 0.001
No	233	37.6	34	30.7	72	29.4	34	53.4	93	46.4	
Missing/not applicable	221	45.5	80	62.6	76	45.1	24	28.9	41	35.6	
Ever tested for HIV	226	38.7	79	56.8	101	48.7	18	24.8	28	16.8	< 0.001
Does not know how HCV is transmitted	442	72.8	99	71.8	130	61.9	68	93.6	145	76.9	< 0.001
Number of days used drugs in last 3 months											
0–30	289	55.3	41	42.5	93	50.2	45	71.7	110	66.5	< 0.001
31–60	132	18.8	22	13.1	50	20.5	18	22.0	42	21.1	
61–90	163	25.9	67	44.4	65	29.4	9	6.2	22	12.3	
Number of sexual partners in last 3 months											
0	235	41.2	29	24.2	15	9.6	26	32.7	165	91.5	< 0.001
1	192	34.7	86	71.0	69	29.1	37	51.2	0	0.0	
2	53	6.9	9	2.7	40	18.0	4	6.9	0	0.0	
3+	92	14.4	6	2.0	81	42.4	5	9.2	0	0.0	
Missing/not applicable	12	2.9	0	0.0	3	0.9	0	0.0	9	8.5	
After using drugs, have more sexual activities											
Yes	189	30.4	32	25.3	131	62.5	25	39.7	1	0.1	< 0.001
No	219	38.9	98	74.7	74	36.2	47	60.3	0	0.0	
Missing/not applicable	176	30.7	0	0.0	3	1.3	0	0.0	173	99.9	
Had group sex											
Yes	32	4.5	2	1.6	30	13.4	0	0.0	0	0.0	< 0.001
No	376	65.1	128	98.4	175	86.0	72	100	1	0.1	
Missing/not applicable	176	30.5	0	0.0	3	0.6	0	0.0	173	99.9	
Had sex with a person of the same sex											
Yes	81	12.5	6	4.0	71	35.7	4	5.9	0	0.0	< 0.001
No	329	57.2	124	96.0	136	64.2	68	94.1	1	0.1	
Missing/not applicable	174	30.3	0	0.0	1	0.1	0	0.0	173	99.9	
Had sex in exchange for money, goods, drugs											
Yes	84	12.9	6	3.8	76	38.5	2	2.2	0	0.0	< 0.001
No	327	56.7	124	96.2	131	60.9	70	97.8	2	0.3	
Missing/not applicable	173	30.4	0	0.0	1	0.6	0	0.0	172	99.7	
Paid someone with money, drugs, goods, in exchange for sex											
Yes	58	8.7	11	4.6	43	23.1	3	3.9	1	0.1	< 0.001
No	352	60.7	119	95.4	164	76.7	68	93.3	1	0.2	
Missing/not applicable	174	30.6	0	0.0	1	0.2	1	2.8	172	99.7	
Been forced to have sex with someone											
Yes	56	8.1	5	6.6	47	18.7	3	4.8	1	0.1	< 0.001
No	350	60.8	125	93.4	157	80.1	67	89.6	1	0.2	
Missing/not applicable	178	31.2	0	0.0	4	1.1	2	5.6	172	99.7	
Did a household member go to prison											
Yes	176	28.2	50	41.4	62	20.7	21	26.7	43	23.6	< 0.001
No	404	71.3	78	57.6	145	78.8	50	72.7	131	76.4	
Missing/not applicable	4	0.5	2	1.1	1	0.5	1	0.6	0	0.0	
Adolescent depression rating scale, median (IQR)	584		130	3.0 (1.0, 6.0)	208	4.0 (2.0, 6.0)	72	3.0 (0.0, 4.0)	174	2.0 (1.0, 4.0)	< 0.001
Psychotic experience (CAPE), median (IQR)	584		130	8.0 (7.0, 9.0)	208	9.0 (8.0, 11.0)	72	8.0 (8.0, 9.0)	174	9.0 (7.0, 10.0)	0.003

Note: All percentages are weighted percentages accounting for the RDS sampling

**Table 5:** Summary of different profiles of HIV exposure in the population sample (*n* = 584)

	Profile 1, high multiple risks	Profile 2, high sexual risks	Profile 3, moderate risks	Profile 4, low risks
*N* (%)	130 (22%)	208 (36%)	72 (12%)	174 (30%)
HIV exposure profile	**Sexual- and drug use-related risk behaviors** This group include most of those engaged in sharing needles, both receptive (9/11) and distributive (10/11) of the entire sample 74% with inconsistent condom use HIV rate, 16%	**Sexual risk behaviors** Few of the YPUD with receptive (2/11) and distributive syringe sharing (1/11) of the entire sample 65% with inconsistent condom use HIV rate, 6%	**Sexual risk behaviors** 64% with inconsistent condom use Never injected drugs. HIV rate, 3%	**No risk identified** 96% never injected 91% never had sex and none reporting inconsistent condom use HIV rate (1%) (Only one YPUD found positive)
Population characteristics	Female, 51% Median age, 23 (21.0, 24.0) HCMC, 63.4% Unstable housing and residential registration document often missing	All transgender and most of the people who have sex with someone of the same sex Median age, 22 (20.0, 23.0) Haiphong, 46.3% More often living alone	Male, 87% Median age, 20 (18.0, 21.0) Living mostly with their family	Male, 88% Median age, 17 (16.0, 19.0) Living mostly with their family
Drug use behaviors	Frequent experience of heroin and methamphetamines Most frequent current drug use	Nearly all have a history of methamphetamines use and most of cannabis, ecstasy, and ketamine use many reporting current regular use of methamphetamines and alcohol	Limited experience of methamphetamines Low level of current drug use	Experienced drugs at a younger age, mainly cannabis but also methamphetamines (67%) Lowest level of current drug use
Sexual behaviors	Inconsistent condom use mainly with their own regular partner	Multiple sexual partners, group sex, frequent drug use during sexual intercourse, More often paid for sex, were paid for sex, forced to have sex with someone	Those sexually active have most often a regular/unique partner	
Other	Many of them orphaned Experience of treatment for drug use and HIV test in the past	Parents less often living together More often regular income More often depressed Have a higher median score of psychotic experience	Poorest knowledge on HCV transmission	Higher median score of psychotic experience

## Discussion

Among this sample of YPUD Methamphetamine is often the first drug used, and it is nearly exclusively inhaled. Cannabis is the second drug of choice, followed by heroin, which is mainly injected. Polysubstance use is common, frequently associated with alcohol use. Methamphetamine use is a major concern, being associated with increased sexual risk behaviors [7, 16, 17] and psychiatric disorders, including depression, psychosis, and suicide [18].

The HIV rate among this sample of YPUD is alarming but mainly located in HCMC, particularly among female YPUD, and among people reporting having sex with someone of the same sex. However, overall, risks of HIV transmission through drug use appeared to be low, with a limited number of participants injecting drugs and with low needle/syringe sharing, mainly also located in HCMC. However, although YPUD reporting drug injection represent just 15% of the total sample in our survey, they account for 51% of all HIV-positive cases. According to the most recent Integrated Biological and Behavioral Survey (IBBS) data from HCMC (2013), around 20% of injecting drug users were still sharing needle/syringe (data for the 6 months prior to interview). It is noteworthy that in HCMC, this rate has remained very high over time compared to previous IBBS, in contrast with other provinces where it has decreased [19]. However, it must be pointed out that in our study, the recruitment strategy may have influenced the sample profile, as the use of peer recruitment in HCMC was the highest compared with the 2 other cities and may have included more long-term injectors known to the peer recruiters (median age of the population sample in HCMC is higher compared to Hanoi and Haiphong). However, it suggests that in this province, interventions to prevent HIV transmission should target first YPUD injecting drugs, highly exposed to HIV due to injection practices in the context of polydrug use and high-risk sexual behavior.

Considering the respondents’ poor knowledge of their own HIV sero-status and the frequency of unprotected sex, the data showed that sexual intercourse is an important HIV-transmission vector in our YPUD sample. Just half of the HIV-positive YPUD reported ever injecting drugs. A gradual switch has been observed over the past 10 years in Vietnam from drug injection-related HIV transmission to sexual transmission with a higher proportion of women HIV-infected [5]. According to the data collected from the Ministry of Health of Vietnam in 2013 in different provinces, 4% of people injecting drugs infected with HIV were aged less than 20, and while needle sharing among PWID was low, sexual risk as measured by inconsistent condom use was high, especially with regular partners [19]. It emphasizes the need for specific and early interventions targeting young people initiating drug use, and particularly sexual risk behaviors associated with drug use.

In our sample, a large proportion of YPUD was suffering from depression and many reported psychotic symptoms. But they also often reported that they often used methamphetamine to cope with sadness and loneliness, emphasizing the complexity of the relationship between mental health and drug use. Many other factors may be related to impaired mental health, including stigma associated with HIV, drug use, homosexuality or transgender status, social impairment, dysfunctional family, and sexual abuse. Many YPUD reported a need for help, particularly mental health support. Thus, psychiatric intervention is crucial, as part of a comprehensive approach but should be designed to reach the needs of this young drug-using population. Mental health disorders play a critical role in HIV acquisition, increasing the risk of HIV acquisition by four to ten-fold and leads to negative health outcomes at each step in the HIV care continuum [20]. Programs targeting people who use drugs are frequently not designed to respond to overlapping vulnerabilities of young people who use drugs, which requires responses that may go beyond the harm reduction programs that are recognized as effective for adults [1]. The number of stimulant users who are seeking treatment is usually extremely low in comparison to the number of individuals with opioid use disorders, due to the lack of a medical model of treatment that includes medication in combination with psychosocial interventions [21]. Innovative interventions, via task-shifting community-based and stepped-care interventions, adapted to the existing system of care, particularly in low-middle-income countries are needed [20, 22]. Peers may play a crucial role [23–25].

The population of YPUD 16–24 is clearly not homogeneous with differences according to their age, the city in which they live, the group to whom they belong (women, MSM/transgender…). One very high-risk group has been identified that include more females, more often HIV-infected, living in precarious situations in HCMC, using heroin and methamphetamines, engaging in unsafe sexual practices with their regular/unique partner. HIV transmission through sexual intercourse with a regular partner who himself uses drugs cannot be excluded [26], and partners of the HIV-infected women in our population sample are probably themselves drug users and HIV-infected.

The second risk group for HIV transmission is identified through high-risk sexual practices. This group reports more multiple partners, commercial sex but also sex abuse, and includes most of the MSM and all transgender people. Methamphetamine and alcohol are more regularly used and are often associated with sexual activity. They are more exposed to not only mental health impairment through regular methamphetamine and alcohol use [18] and hyper-stigmatization due to their MSM and transgender status [27, 28], but also sexual abuse and dysfunctional family relationships.

The third profile describes those with low transmission risk which centers, only on sexual unprotected sex but with a unique partner. This group presents a low level of drug use and lives in a stable situation.

The fourth profile can be currently considered as nearly free of any risk of HIV transmission: these YPUD nearly never injected drugs and very few had sex, none with inconsistent condom use. They are the youngest YPUD and are mainly using cannabis. It is difficult to know if this group of YPUD is really different from the other groups or the same population but at an earlier stage of drug use initiation. A longitudinal survey would help to better understand this aspect. They present a high median score of psychotic experience that could be related to their regular cannabis use [29] or regular use of methamphetamine for a few of them but with a higher vulnerability due to their young age [30, 31].

These two last groups have the poorest knowledge of HIV/HCV transmission suggesting that wide information on HIV/HCV transmission and simple harm reduction messages should be widely disseminated, including at the school level.

Despite the limited size of the population sample which limits conclusions, the heterogeneity of HIV exposure profiles in our study probably reflects the real heterogeneity of YPUD population, at least in the urban environment in Vietnam. This dimension needs to be taken into account when designing interventions for this population.

There are several public health implications resulting from this study (see Table 6).

**Table 6:** suggested interventions according to the YPUD profile

	Profile 1, high multiple risks	Profile 2, high sexual risks	Profile 3, moderate risks	Profile 4, low risks
*N (%)*	130 (22%)	208 (36%)	72 (12%)	174 (30%)
Suggested interventions	Peer and CBO evaluation of YPUD needs according to sex, age, city, key group Develop training and empowerment for peer and CBO Information-education-communication			
	*Comprehensive approach including:* Harm reduction Social/administrative support Quick access to and retention on methadone Access and adhere to HIV treatment Psychosocial interventions for meth users Mental health interventions Family intervention Repeated HIV/HCV screening for YPUD and regular partners	Harm reduction targeting sexual risk behaviors including PrEP Psychosocial interventions for meth/alcohol users Mental health interventions Family intervention Repeated HIV/HCV screening	Information on HIV/HCV transmission and harm reduction Control/reduce frequency of meth use	Prevent mental health complications of meth/cannabis use Lowering meth/cannabis use, delay initiation for new drugs Information on HIV/HCV transmission and harm reduction No other specific HIV prevention measures

Given the limited resources of health care staff in the mental health/addiction field in Vietnam, in contrast to the considerable expertise of peers, CBO and peers should be empowered through information, training, constant support, and supervision from professionals. It would allow them to appropriately assess the needs of YPUD according to the local context and population and define and implement adapted interventions. These interventions should include, for all YPUD, increased knowledge on HIV, HCV, and drugs and information on mental health and intervention related to family. Mental health interventions are critical considering the rate of mental health disorders in our population sample, their multiple social impairments including deteriorated family relationships, stigmatization of their status, and history of sexual abuse. Psychosocial interventions are necessary for all regular drug users, particularly frequent methamphetamine users. Repeated testing for HIV/HCV should be proposed to those with the most at-risk profiles (profiles 1 and 2) and their regular partners (profile 1). Regarding harm reduction interventions, it should be extended to target methamphetamine use, drug injection (needle/syringe and paraphernalia exchange programs), polydrug use and unsafe sex for profile 1 and unsafe sex for profile 2 with interventions adapted to drug use in a sexual context (chemsex) including pre-exposure prophylaxis (PrEP), and taking into account excessive alcohol use. For profile 3, harm reduction should focus on methamphetamine use. For the youngest ones, the question of the potential damages related to early and regular drug use has to be raised. The consequences of early drug use are not the same at age 16 or 24 years and efforts should focus on interventions to reduce the level of drug use and delay the initiation of new drugs, particularly methamphetamines, but also cannabis. For the profile 4 population, targeting HIV prevention is probably less crucial than preventing mental health complications. Above all, quick and easy access to medical treatment (methadone for all opioid-dependent YPUD and antiretroviral treatment for all HIV-positive YPUD) should be offered, including support for administrative procedures. In our population sample, YPUD in HCMC clearly require an immediate and comprehensive intervention including most of the aspects described above.

There are several limitations to this study. The data collected on sexual and injection risk behaviors were based on self-report. As there may be stigma related to reporting specific injection and sexual risk behaviors, particularly among YPUD, there may be underreporting of these risk behaviors. However, the problem associated with the validity of self-reports by drug-using individuals has already been widely documented [32, 33] but it is very difficult to assess risk-related practices in ways other than through self-reporting. However, self-report of risky practices in our survey is congruent with data collected at the national level through IBBS. Drug use was assessed through self-report and urine testing. Results of urine tests may lead to underestimation of substance use, particularly for YPUD with irregular use of drugs, the tests reflecting only recent intake (1 to 3 days for methamphetamine use and 1 to 3 days for occasional cannabis use). Ketamine was not screened in our survey. The study was cross-sectional in nature and does not permit causal inference. Another point is that due to the difficulty in reaching this population, the RDS strategy had to be adapted at some point to enlarge recruitment through peers. It may also have had an impact on the representativeness of our population sample. As the YPUD in our study originated from the 3 largest cities in Vietnam, they may not be fully representative of YPUD from all provinces, including the rural areas. This may be particularly true in HCMC where most of the HIV-positive YPUD are located and the highest rate of risk behaviors identified. Considering that HIV-positive YPUD aware of their status may adapt their behavior to avoid HIV transmission, only data from YPUD who reported being HIV negative or not knowing their HIV status may have been recruited. As three fourths of the HIV-positive YPUD reported that they never had been screened for HIV before or had been screened but did not know their status, they were finally all included in the data analysis.

## Conclusions

In our population sample of YPUD aged 16 to 24 years, methamphetamine is the first drug used, nearly exclusively inhaled, followed by cannabis and heroin, injected; HIV prevalence is high, most of the HIV-positive YPUD being located in HCMC, particularly among injecting drug users; and mental health problems are very common and require special attention. HIV exposure among YPUD in these three major cities of Vietnam is important but varies considerably according to age, city, and population group to whom they belong. Four profiles were identified: one with high sexual and drug-related risk behaviors and a high HIV rate (16%), one with sexual risk and a 6% HIV rate, the third with low risk, mainly sexual and a 3% HIV rate, and the last one with no current HIV-infection risk and a less than 1% HIV rate. Interventions need to be designed accordingly with an immediate and special attention for YPUD injecting drugs, who are reporting syringe/needle sharing, unsafe sex, and polydrug use, in order to prevent them from getting HIV and transmitting to others. Our results highlight also the need to address sexual-related risks to prevent HIV and develop adapted interventions including PrEP. Overall, these interventions need to be stratified, starting from basic and universal preventive measures to specific harm reduction/therapeutic interventions, integrated in a holistic approach according to the needs of the groups of YPUD.

## Data Availability

The datasets used and/or analyzed during the current study are available from the corresponding author on reasonable request.

## Abbreviations

ADRS: Adolescent Depression Rating Scale
CAPE: Community Assessment of Psychic Experience
CBO: Community-based organizations
FSW: Female sex worker
HCMC: Ho Chi Minh City
HCV: Hepatitis C virus
HIV: Human immunodeficiency virus
IBBS: Integrated Biological and Behavioral Survey
MCA: Multiple correspondence analysis
MSM: Men who have sex with men
PLHIV: People living with HIV
PrEP: Pre-exposure prophylaxis
PWID: People who inject drugs
RDS: Respondent-driven sampling
UNODC: United Nations Office on Drugs and Crime
VND: Vietnamese Dong
WHO: World Health Organization
YPUD: Young people who use drugs
